# Dissemination of different sequence types lineages harboring *bla*_CTX-M-15_ among uropathogenic *Escherichia coli* in Kerman, Iran

**DOI:** 10.22038/ijbms.2020.47520.10940

**Published:** 2020-12

**Authors:** Davood Kalantar-Neyestanaki, Shahla Mansouri, Mohammad Reza Kandehkar Ghahraman, Fatemehalsadat Tabatabaeifar, Zahra Hashemizadeh

**Affiliations:** 1 Medical Mycology and Bacteriology Research Center, Kerman University of Medical Sciences, Kerman, Iran; 2 Department of Microbiology and Virology, School of Medicine, Kerman University of Medical Sciences, Kerman, Iran; 3 Student Research Committee, Kerman University of Medical Sciences, Kerman, Iran; 4 Facultad de Ciencias Químicas, Universidad Autónoma de Nuevo León, UANL, San Nicolás de los Garza, Mexico; 5 Centro de Investigación en Biotecnología y Nanotecnología, Facultad de Ciencias Químicas, Parque de Investigación e Innovación Tecnológica, Universidad Autónoma de Nuevo León, Apodaca, Mexico; 6 Department of Bacteriology and Virology, School of Medicine, Shiraz University of Medical Science, Shiraz, Iran

**Keywords:** Antimicrobial resistance, Biofilm, Escherichia coli, Multilocus sequence typing, Urinary tract infections

## Abstract

**Objective(s):**

*Escherichia coli* is one of the most important causes of urinary tract infections (UTIs). The aim of this study was to determine antimicrobial resistance, resistance and virulence genes; phylogenetic groups and identify the epidemiologic features of uropathogenic *E. coli* (UPEC) isolates by multilocus sequence typing (MLST).

**Materials and Methods:**

One hundred isolates of *E. coli* from inpatients with UTIs were collected in Kerman, Iran. Antimicrobial susceptibility testing, ESBLs, AmpC production and biofilm formation were performed by phenotypic methods. Phylogenetic groups, resistance and virulence genes were detected. Molecular typing of isolates was performed by MLST.

**Results:**

In this study, 76% of isolates were multidrug-resistant. The *bla*_CTX-M-15_ and *bla*_TEM_ were the dominant ESBL-encoding gene. Among 63 ciprofloxacin-resistant isolates, the frequency of *qnrS* (15.8%), *qnrB* (9.5%), and *aac* (6’)-*Ib* (25% ) genes was shown. Fifty-five present of isolates were classified as week biofilm, (14%) moderate biofilm, and (5%) strong. The predominant phylogenetic group was B2 (3) . The prevalence of virulence genes ranged *fimH* (93%), *iutA* (66%), *KpsmtII* (59%), *sat* (39%), *cnf* (28%) and *hlyA* (27%). According to MLST results, 14 sequence types (ST) including ST-693, ST-90, ST-101, ST-1664, ST-2083, ST-131, ST-4443, ST-744, ST-361, ST-405, ST-922, ST-648, ST-5717and ST-410 were detected, indicating a high degree of genotypic diversity.

**Conclusion:**

We identified a high frequency of the ST131 clonal group among UTIs. These data show an important public health threat, and so further studies to control the dissemination and risk factors for acquisition of the ST131 clonal group and other STs are needed to make effective control.

## Introduction

*Escherichia* *coli* (*E. coli*) is one of the most important causes of urinary tract infections (UTIs), neonatal meningitis, bacteremia, and community- and hospital- acquired infections. According to epidemiological data, almost 20% of men and 50-60% of women in their life may suffer from UTIs (1). Different antibacterial agents such as fluoroquinolones, aminoglycosides, trimethoprim/sulfamethoxazole, and β-lactams are recommended for treatment of UTIs (2, 3,4*, *5*)*. The frequent use of antibiotic in treating leads to drug resistance, which results in the spread of multidrug-resistant (MDR) isolates (6). One of the most common causes of resistance to antibiotics in *E. coli* is the production of extended spectrum beta lactamase (ESBLs) that causes bacterial resistance to the third, and fourth-generation cephalosporins, and monobactams, especially in hospital-acquired infection. ESBL-producing uropathogenic *E. coli* (UPEC) are often MDR to different classes of antibiotics including aminoglycosides, and fluoroquinolones. Resistance to these agents causes delays in suitable therapy with subsequently increasing morbidity and mortality (2, 6). UPEC has several virulence factors such as adhesion, fimbria (*fimH*), toxins (*hlyA, sat, cnf*), aerobactin-mediated iron uptake (*iutA*), capsular polysaccharide (*KpsmtII*) and biofilm formation that lead to colonization of host mucosal surface, damage and invasion to host tissue (7, 8). It also makes colonization in the bladder and causes cystitis. Studies have shown that the most important stage of infections is attachment to the host tissue (9). The ability of *E. coli* to adhere to uroepithelial cells is a virulence factor. Fimbria is the most important pathogenic factor in UTI; also *E. coli* has the ability to form biofilm in the urinary tract system, especially in the bladder (9). Recently, a relation between presence of virulence genes and *E. coli* phylogenetic characteristics has been reported (3). Gram negative bacteria have the ability to move through ureters to kidney and cause pyelonephritis (10). Biofilms are small colonies of bacteria surrounded by extracellular matrix that helps to the assembly and attachment of them to the tissue, causing tissue damage. More than 60% of human infections are due to biofilm. Biofilm causes bacteria resistance to the host immune system and antimicrobial agents (11). 

Recent studies explained a relation between presence of virulence genes and *E. coli* phylogenetic characteristics. Phylogenetic groups have been determined to four major phylogenetic groups (A, B1, B2 and D) and seven subgroups A0, A1, B1, B2 (2), B2 (3), D1 and, D2 in UPEC strains (3, 12, 13). Extraintestinal pathogenic *E. coli* (ExPEC) isolates usually belong to phylogenetic groups B2, and D, and commensal *E. coli *isolates belong to phylogenetic groups A and B1 (13). Moreover, pathogenic extraintestinal isolates acquire specific virulence factors conferring their ability to pathogenic potential (14). Many various typing methods have been applied for identification of bacterial infection sources, as well as the prevention and control of the spread of infections (15, 16). Multilocus sequence typing (MLST) is a method for molecular typing of bacteria. This method is based on 450-500 bp fragments of seven housekeeping genes loci, and the result of allelic profile is based on sequence type (ST) via a database and can be compared genetic relatedness between isolates (15, 17,18). The aim of this study was to determine antimicrobial resistance profile, resistance and virulence genes, phylogenetic groups and the epidemiologic features of UPEC isolates by MLST method.

## Materials and Methods

***Population and bacterial isolates***

Totally, 100 isolates of *E. coli* from inpatients with UTIs from June 2017 to June 2018 were isolated in Kerman, Iran. All isolates were identified by standard bacteriological methods (19) .

The study was approved by the Ethics Committee of Kerman University of Medical Sciences (IR.KMU.REC. 1394.327).

***Antibacterial susceptibility testing, screening of ESBL and AmpC producer isolates***

Antimicrobial susceptibility testing of all isolates to gentamicin (GM, 10 µg), amikacin (AK, 30 µg), pipracillin/tazobactam (PIT, 100/10 µg), trimethoprim/sulfamehoxazole (SXT, 5 µg), imipenem (IMP, 10 µg), amoxicillin/clavulanicacid (AUG, 30/10 µg), ciprofloxacin (CIP, 5 µg), nalidixic acid (NA, 30 µg), ceftazidime (CAZ, 30 µg), cefepime (CPM, 30 µg), and cefotaxime (CTX, 30 µg) was performed using the disk diffusion method according to the clinical and laboratory standard institute (CLSI 2018) guidelines. *Pseudomonas aeruginosa* ATCC 27853 and *E. coli* ATCC 25922 were used as control bacteria in antibiogram testing (20). MDR isolates were distinct as described by previous study (21). ESBLs producing isolates were determined according to CLSI recommendations. AmpC disk test was used for detection of AmpC producing isolates (22).

***Detection of resistance genes by polymerase chain reaction***

The boiling method was used for preparation of DNA template for polymerase chain reaction (PCR) (23) .The oligonucleotide primers were used for identifying the *bla*_CTX-M _group 1-4, *bla*_TEM_, *bla*_SHV_, *bla*_OXA_, *bla*_PER_, *bla*_KPC_ and *bla*_NDM __,_*qnrs*, *qnrA*, *qnrB*, *rmtA*, *rmtB*, *rmtC*, *rmaA* (24,25, 26). PCR amplification was set up in a total volume of 25 μl containing 0.5 µl of each primer (10 pM), 12.5 µl of DNA Polymerase Master Mix RED (Ampliqon, Co, Denmark), 1 µl of DNA and 10.5 µl of water in Biometra PCR Thermal Cycler (Biometra, Germany) under the following conditions: initial denaturation at 95 ^°^C for 5 min followed by 30 cycles of denaturation at 95 ^°^C for 1 min, annealing at 55-64 ^°^C for 1 min (Table 1), extension at 72 ^°^C for 1 min and final extension at 72 ^°^C for 5 min. In the end, PCR products were electrophoresed on 1.5% agarose gel in 0.5 TBE buffer (Tris, EDTA, Boric acid).

***PCR products sequencing***

The *bla*_CTX-M _positive amplification was sequenced in Macrogen, Co, South Korea. Then, the acquired nucleotide sequences were compared using online Basic Local Alignment Search Tool (BLAST) software (www.ncbi.nih.gov/BLAST program), and established as *bla*_CTX-M-15 _variant. 

***PCR method for the detection of virulence genes***

All primers were the same as those used in previous studies (3, 12, 27). The PCR mixtures (25 µl) contained 1 µl of DNA, 12.5 µl of PCR master mix (Ampliqon, Inc, Co, Denmark), 0.5 µM (10 pM) of each primer and 10.5 µl of water (DNase and RNase free water). PCR amplification was comprised of the following three steps: heating at 95 ^°^C for 5 min; 30 cycles of denaturation at 95 ^°^C for 1 min, primer annealing at 58-63 ^°^C for 1 min and extension at 72 ^°^C for 1 min, followed by a final extension step of 72 ^°^C for 5 min. Amplicons were revealed by electrophoresis on a 1.5% agarose gel, and photographed using a UV transillumination imaging system.

***Biofilm assay***

We analyzed the ability of the UPEC isolates to produce the biofilm according to the protocol described by Stepanović *et al.* (28). The Positive control for the assay was *P. aeruginosa* strain PAO1 and the culture medium without bacteria was used as the negative control.

***Phylogenetic grouping***

The distribution of phylogenetic groups in 100 UPEC isolates was determined by phylotyping PCR approach described by Clermont and colleagues (12). The results of these three amplifications allowed the classification of *E. coli *isolates into one of the major phylogenetic groups: A, B1, B2 or D and sub phylogenetic A0, A1, B1, B2 (2), B2 (3), D1, D2 (29).

***Molecular typing by MLST***

PCR amplification and sequencing of seven housekeeping genes (*adk, fumC, gyrB, icd, mdh, purA* and *recA*) were performed following the protocols specified at the *E. coli* MLST website (http://mlst.warwick.ac.uk/mlst/dbs/Ecoli). All the primer sequences of seven genes are available at http://mlst.warwick.ac.uk/mlst/dbs/Ecoli/documents/primersColi_html.

 The 50 μl of amplification reaction mixture comprised 1 µl of each primer (10 pM), 25 µl of DNA Polymerase Master Mix RED (Ampliqon, Co, Denmark), 2 µl of DNA and 21 µl of DNase and RNase free water in FlexCycler PCR Thermal Cycler (Analytik Jena, Germany). The reaction conditions were an initial denaturation step at 95 ^°^C for 5 min, followed by 30 cycles of the following conditions: denaturation at 95 ^°^C for 1 min, 1 min primer annealing at 60-63 ^°^C, and extension at 72 ^°^C for 2 min, with a final extension step at 72 ^°^C for 5 min.

Sequencing of the PCR products was performed using the services of Macrogen (Macrogen, South Korea). Allele numbers for seven gene fragments of each isolate were obtained by comparing with corresponding allele available in MLST *E. coli* database (http://mlst.warwick.ac.uk/mlst/dbs/*Ecoli*), and ST of each isolate was determined by combining seven allelic profiles.

***Statistical analysis***

The SPSS software version 22.0 (IBM, Armonk, NY, USA) was used for data analysis. *P- value* of ≤0.05 was considered as statistically significant.

## Results

Total of 100 UPEC isolates were collected from different ward including emergency (39%), pediatric (18%), surgery (7%), rheumatology (10%), labor (5%), urology (4%), neonatal (3%), transplant (2%), ICU (2%), CCU (2%), ENT (1%), and other wards (9%) from Afzalipour, Shafa and Bahonar hospitals in Kerman. The age of the study group ranged from 1 to 90 years. Totally, 65% and 35% of isolates were detected in urine of female and male patients.

***Antimicrobial susceptibility testing***

The antimicrobial susceptibility results showed that 100% of isolates were susceptible to imipeneme (IMP). The resistance patterns amongst the isolates were observed for amoxicillin/clavulanic acid (AUG, 75%), trimethoprim/sulfamehoxazole (SXT, 75%), nalidixic acid (NA, 75%), ciprofloxacin (CIP, 63%), cefotaxime (CTX, 56%), cefepime (CPM, 56%), ceftazidime (CAZ, 56%), pipracylin/tazobactam (PIT, 27%), gentamaycin (GM, 19%) and amikacin (AK, 6%). The rate of MDR isolates were 76%. ESBL and AmpC-β-lactamases were observed in 55 and 3.5% of isolates, respectively. There were significant differences in resistance to the aforementioned antibiotics and ESBL production, except GM among the UTI isolates (Table 1).

***Distribution of antibiotic resistant genes***

ESBLs genes detected by PCR method was *bla*_TEM _(41, 74.5%), *bla*_CTX-Mgroup1_ (41, 74.5%), and *bla*_SHV_, *bla*_OXA _(8, 14.5%). Among 63 ciprofloxacin-resistant isolates, ﬂuoroquinolones resistance genes including *qnrS*, *qnrB, *and* aac(6’)-Ib* were detected with the frequency of (15.8%), ( 9.5%) and (25%), respectively , which were confirmed by PCR sequencing. The efflux pump genes *oqxA* and *oqxB* were detected in 2% of isolates. Other resistance genes were not detected in any of the isolates tested.

***Distribution of phylogenetic groups***

The predominant phylogenetic group was B2 (3) (30%), followed by D2 (22%), B1 (15%), A1 (13%), A0 (8%), D1 (8%), and B2 (2) (4%). Relationship between phylogenic groups, antibiotics resistance and virulence genes was shown in Table 2.

***Distribution of virulence-associated genes and biofilm***

The prevalence of virulence genes was as follow: *fimH *(93%), *iutA *(66%),* KpsmtII *(59%), *sat *(39%),* cnf *(28%) and *hlyA *(27%). Among the detected biofilm formation, 55% of isolates were classified as week biofilm, 14% moderate biofilm, 5% strong and 26% non-biofilm. There were not significant differences in resistance to the aforementioned antibiotics and production of biofilm, except CAZ (P=0.03) among the UTI isolates (Table 1).

***STs created via MLST and CC identification***

**Figure 1:**
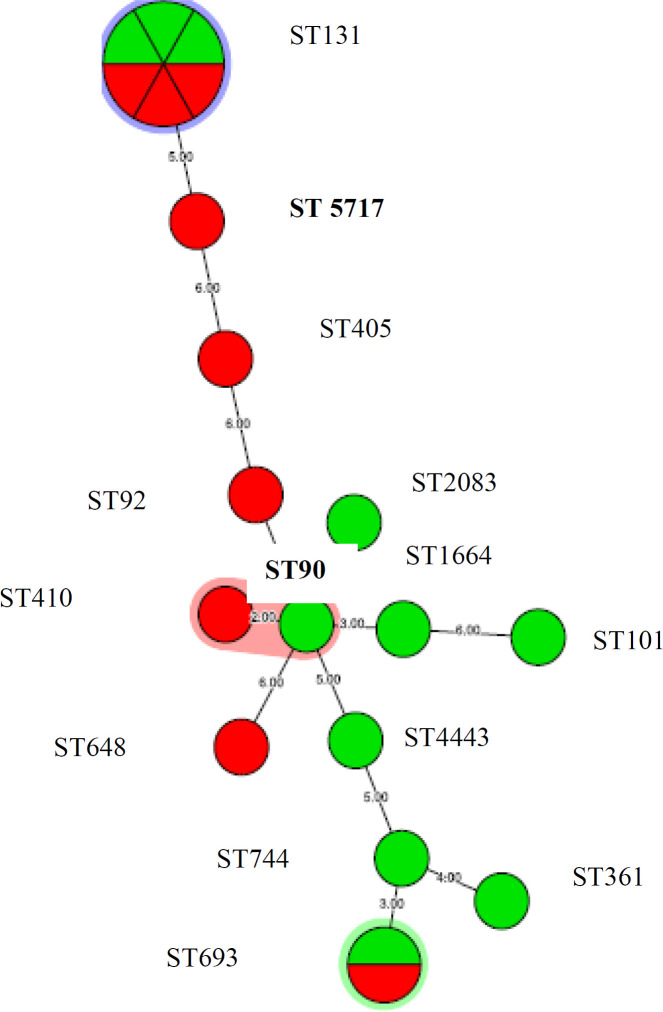
Minimum spanning tree (MST) in 20 blaCTX-M-15 positive isolates. Red: Shafa hospital, Green: Afzalipour hospital

MLST analysis of the 20 ESBLs and *bla*_CTX-M-15 _positive isolates was studied. The The Based Upon Related Sequence Types (eBURST) algorithm used in this study determined the 14 STs into 5 CCs (CC131, CC648, CC101, CC405, CC23), indicating a high degree of genotypic diversity. Among these STs, ST131 was predominant (6 isolates, 30%), followed by ST-693 isolates (2, 10%) (Table 3). The five most common CCs were CC131 (n=6), CC23 (n=2), CC648 (n=1), CC101 (n=1) and CC405 (n=1). ST-131 (CC131) was the most prevalent CC, comprising 6 (30%) isolates. Among the three clonal complexes, CC131 was the largest, containing 6 isolates, consisting of ST131, in ST131 Cplx (Sequence types and complex). The second largest clonal complex was CC23, which contained 2 isolates, consisting of ST90 and ST410 in ST23 Cplx. The minimum clonal complex was CC648, consisting of ST648 in ST648 Cplx, CC101 consisting of ST101 in ST101 Cplx, CC405 consisting of ST405 in ST405 Cplx and rest of STs without a clonal complex (Table 4). The isolates from Shafa hospital specimens predominantly belonged to 9 different STs (ST-693, ST-90, ST-101, ST-1664, ST-2083, ST-131, ST-4443, ST-744, ST-361), whereas Afzalipour hospital isolates belonged to 7 different STs (ST-405, ST-131, ST-922, ST-648, ST693 ST-5717and ST-410). In both hospital, ST-131 predominated and was responsible for 30% of hospital infections. MLST results are summarized in Tables 3 and 4 and minimum spanning tree (MST) in 20 isolates UTI *bla*_CTX-M15 _was shown in Figure 1**.**

**Table 1:** Relationship between antibiotics resistant and production of biofilm and ESBL

**Presence of ESBL, Biofilm**	**Resistant of antibiotics (No)**										
	**AUG**	**SXT**	**NA**	**CTX**	**CPM**	**CAZ**	**IMP**	**AK**	**PIT**	**CIP**	**GM**
**ESBL**	70	75	73	56	53	55	4	6	27	63	19
**P-value**	0.02	0.03	0.0001	0.0001	0.0001	0.0001	0.02	0.03	0.001	0.002	0.1
**Biofilm**	52	58	57	46	44	47	3	5	22	48	16
**-value**	0.8	0.5	0.2	0.2	0.08	0.03	0.7	0.01	0.3	0.7	0.7

AUG: amoxicillin-clavulanic acid; SXT: trimethoprim/sulfamehoxazole; NA: nalidixic acid; CTX: cefotaxime; CPM: cefepime; CAZ: ceftazidime; IMP: imipenem; AM: amikacin; PIT: piperacillin/tazobactam; CIP: ciprofloxacin; GM: gentamicin; ESBLs: Extended spectrum beta lactamase

**Table 2:** Relationship between phylogenic groups and antibiotics resistant and virulence genes

**Antibiotics & virulence genes**	**Phylogenic groups**							
	**A0**	**A1**	**B1**	**B2(2)**	**B2(3)**	**D1**	**D2**	**-value**
**AUG**	7	8	12	3	20	6	14	0.8
**SXT**	8	9	13	3	23	5	14	0.4
**NA**	7	11	13	1	24	3	14	0.02
**CTX**	3	9	9	1	21	4	9	0.2
**CPM**	3	9	7	1	21	3	9	0.1
**CAZ**	3	8	10	1	20	4	9	0.3
**IMP**	1	0	1	0	2	0	1	0.8
**AK**	0	1	1	0	2	1	1	0.9
**PIT3**	3	5	4	1	8	1	5	0.8
**CIP**	7	11	13	2	19	3	8	0.008
**GM**	2	2	6	0	8	1	0	0.06
***hlyA***	0	1	3	2	10	3	8	0.1
***fimH***	7	11	15	4	28	7	21	0.7
***iutA***	3	8	8	4	20	6	17	0.2
***cnf***	0	0	4	3	10	2	9	0.02
***kpsMT II***	4	6	2	4	23	6	14	0.001
***sat***	0	2	4	3	15	4	11	0.02

AUG: amoxicillin-clavulanic acid; SXT: trimethoprim/sulfamehoxazole; NA: nalidixic acid; CTX: cefotaxime; CPM: cefepime; CAZ: ceftazidime; IMP: imipenem; AM: amikacin; PIT: piperacillin/tazobactam; CIP: ciprofloxacin; GM: gentamicin

**Table 3:** Distribution of gender, age, hospital, ward, pattern of antibiotics and ST in 20 *bla*_CTX-M-15_ positive isolates

**Number**	**Gender**	**Age**	**Hospital**	**Ward**	**AUG**	**SXT**	**NA**	**CTX**	**CPM**	**CAZ**	**AK**	**PIT**	**CIP**	**GM**	**ST**
	female	26	Shafa	Romatology	R	R	R	R	R	R	S	R	R	R	693
2	female	60	Afzali	Romatology	R	R	R	R	R	R	S	R	R	S	648
3	male	23	Shafa	Surgery	S	R	R	R	R	R	S	S	R	R	131
4	male	55	Afzali	Surgery	R	S	R	R	R	S	S	R	R	R	131
5	female	48	Afzali	Laber	R	R	R	R	R	R	S	R	R	R	131
6	female	32	Afzali	Laber	R	R	R	R	R	R	S	S	R	S	922
7	male	45	Shafa	Other	R	R	R	R	R	R	S	R	R	R	131
8	female	62	Afzali	Other	R	R	R	R	R	R	R	R	R	S	693
9	male	52	Shafa	Surgery	R	R	R	R	R	R	S	S	R	S	1664
10	female	36	Afzali	Laber	R	S	R	R	R	R	S	S	R	S	131
11	female	31	Shafa	Romatology	R	R	R	R	R	R	S	R	R	R	361
12	female	48	Shafa	Emergency	R	R	R	R	R	R	S	S	R	S	90
13	female	17	Shafa	Romatology	R	R	R	R	R	R	S	R	R	S	4443
14	male	67	Shafa	ICU	R	R	R	R	R	R	S	S	R	R	744
15	male	32	Shafa	Surgery	R	S	R	R	R	R	R	R	R	R	131
16	male	30	Shafa	Other	R	R	R	R	R	R	S	S	S	S	2083
17	female	30	Afzali	Other	R	R	R	R	R	R	S	S	R	S	405
18	female	32	Afzali	Other	R	S	R	R	R	R	S	S	R	S	5717
19	male	67	Afzali	Transplant	R	R	R	R	R	R	S	R	R	S	410
20	male	68	Shafa	CCU	R	R	R	R	R	R	S	R	R	S	101

AUG: amoxicillin-clavulanic acid; SXT: trimethoprim/Sulfamehoxazole; NA: nalidixic acid; CTX: cefotaxime; CPM: cefepime; CAZ: ceftazidime; IMP: imipenem; AM: amikacin; PIT: piperacillin/tazobactam; CIP: ciprofloxacin; GM: gentamicin, R: resistant, S: sensitive, ST: sequence type, CCU: Coronary care unit; ICU: Intensive care units

**Table 4:** Distribution of virulence genes, phylogeny groups and ST in 20 *bla*_CTX-M_ positive isolates

**Number**													
	**ST**	**CC**	**Biofilm**	***hlyA***	***fimH***	***iutA***	***cnf***	***kpsMTІІ***	***sat***	***bla***_OXA_	***bla***_TEM_	***qnrB***	**Phylogenic group**
	693	-	W	N	N	P	N	N	N	N	P	N	A0
2	648	ST648Cplx	W	N	P	P	N	P	P	P	P	N	B2(3)
3	131	ST131Cplx	W	N	P	P	N	P	P	N	N	N	B2(3)
4	131	ST131Cplx	N	N	N	P	N	P	N	P	P	N	A1
5	131	ST131Cplx	M	P	P	P	P	P	P	P	P	N	B2(3)
6	922	-	W	N	P	P	N	P	P	N	P	N	B2(3)
7	131	ST131Cplx	W	N	P	N	N	N	N	N	P	N	A0
8	693	-	W	N	P	P	N	N	N	P	P	N	A1
9	1664	-	N	N	P	N	N	N	P	N	P	N	B2(3)
10	131	ST131Cplx	W	N	P	N	N	P	N	N	P	N	B2(3)
11	361	-	W	N	P	N	N	N	N	N	P	N	A1
12	90	ST23Cplx	W	N	P	P	N	N	N	N	P	N	B1
13	4443	-	M	N	P	N	N	N	N	N	P	N	B2(3)
14	744	-	M	N	P	N	N	N	N	N	P	N	B2(3)
15	131	ST131Cplx	W	N	P	P	P	P	P	N	P	N	B2(3)
16	2083	-	W	N	P	P	P	P	P	N	P	N	B2(3)
17	405	ST405Cplx	S	N	P	N	N	N	N	N	N	N	D2
18	5717	-	N	N	P	P	P	P	P	N	P	N	B2(2)
19	410	ST23Cplx	W	N	P	P	N	N	N	N	P	N	A1
20	101	ST101Cplx	W	N	P	N	N	N	N	N	P	P	A0

W: Weak, M: Moderate, S: Strong, N: Negative, P: Positive. CC: Clonal Complex, ST: sequence type

## Discussion

*E. coli* is one of the most common bacterium in UTIs (16). Today, improper use of antibiotics has increased the resistance to various antibiotics (30). In our study, the rate of multidrug resistance among the UPEC isolates was high (76%). The highest resistance was observed against AUG, SXT, and NA (75%) and the lowest resistant was recorded against AK (6%). Also, high susceptibility was found for IMP (100%).

In recent years, MDR , AmpC and ESBL-producing *E. coli* isolates have been increased in different parts of the world, and it has become an emerging public health problem (31, 32). In this study, prevalence of AmpC and ESBL-producing *E. coli* isolates have been reported 3.5%, and 55%, respectively. As previously noted,* bla*_CTX-M _genes were commonly found in large R-plasmids and carry other genes, leading to resistance to other antimicrobial agents such as fluoroquinolones and aminoglycosides (33). In our study, the rate of *bla*_CTX-M_ gene and *bla*_TEM_ were 74.5%. The data in this study confirms previous studies indicating that ESBL-producing *E. coli* isolates and β-lactamase genes such as *bla*_CTX-M _and *bla*_TEM_ have been increased in different parts of the world (34, 35). Four types of plasmid-mediated quinolone resistance (PMQR) (*qnr, aac(6’)-Ib-cr, qepA*, *oqxAB*) have been recognized. This plasmid (PMQR) can confer resistance to multiple agents, including fluoroquinolones and ESBLs genes (36). In this study, the rate of PMQR genes was 52.3%. In our study, significant differences between the antibiotic resistant and ESBL production were detected except GM antibiotics (*P*<0.05). This indicates that ESBL production is important factor in antibiotic resistance. In this study, 43% of isolates were ciprofloxacin resistant and ESBL positive, and we found a statistically significant difference between resistant to ciprofloxacin and ESBL production (*P*=0.002). Also, we showed that fluoroquinolones -resistance genes such as *qnrS *(10, 23.2%),* qnrB *(6, 13.9%), and *aac(6’)-Ib-cr* (25,58.1%) were strongly related with an ESBL-positivity in UPEC. This is in accordance with the results obtained by previous study (37). Biofilm production can protect bacteria from killing activity of host defense mechanisms and antibiotics. As well, it can cause expression of several virulence factors, and increase resistance against antibiotics (38). Characterization of virulence markers and drug resistance of UPEC let the physicians to follow up the development of pathogenicity of strains causing the UTIs and improving obtainable infection control policies (39). The significant differences between the biofilm formation and antibiotic resistance to CAZ (*P*=0.03) and AK (*P*=0.01) were detected. This indicates that biofilm formation can be considered as one of the factors in antibiotics resistance. Also, the prevalence of virulence genes of UPEC isolates were *fimH *(93%), *iutA* (66%),* kpsMTII (59%), sat (39%), cnf (28%), and hlyA (27%). *Therefore, high incidence of virulence genes could be a main causative agent for UTIs in humans. In this study, highly virulence isolates were mostly present in group B2 (3) and D (2) and less virulence strains were present in group A0. As well, we observed a statistically significant association between microbial resistance to NA (*P*=0.02), and CIP (*P*=0.008) and the phylogenic groups. So, the highest antibiotic resistance was observed in group B2 (3). In addition, statistically significant difference was observed between the presence of phylogenic groups and virulence genes of *cnf* (*P*=0.02), *sat *(*P*=0.02), and *kpsMT II* (*P*=0.001)***.*** This indicates that the highest rate of *cnf*, *sat* and *kpsMT II* genes virulence was observed in group B2 (3). 

MLST is the best method for studying molecular epidemiology. The MLST typing determines the diversity and phylogenetic relationships of the isolates, which rely on seven housekeeping genes for each *E. coli *isolate that reflects population structure and evolutionary biology of bacteria. Also, this method provides comparisons between results from various laboratories (40). The portability and reproducibility of MLST will present valuable and significant information about *E. coli *genetic lineage in UTI (15). This study also focused on epidemiological investigation of MLST in the *bla*_CTX-M-15_ UPEC*.* Based on these findings, ST-131 was predominant ST-type in our hospital settings. As well, 14 ST complexes that are associated with UPEC have been recognized; these are ST-693, ST-648, ST-922, ST-90, ST-361, ST-405, ST-101, ST-1664, ST-2083, ST-5717, ST-410 and ST-744 complexes. Based on the MLST method, the isolates of 5 CCs (CC131, CC648, C101, CC405, and CC23) clustered together in the minimum evolution (ME) tree, suggesting their status as genetically exclusive complexes or groups. Among the 13 STs and 5 CCs identified in this study, CC131 was the most common clonal complex, comprising 6 isolates. Based on these explanations, CC131 could represent the main UPEC strains worldwide. CC131 and ST131 are widely disseminated and play main roles in UPEC infection. Previous studies recognized that ST131 was predominant among ESBL producing *E. coli*. In addition, four major ST complexes, including ST-14, ST-69, ST-73, and ST-95 have been identified in UPEC (41). According to previous studies, discrepancy in ST131 biofilm formation is associated with bacterial culture conditions, data cut-offs and definitions used in prevalence studies and clonal diversity within each ST131 collection (42). In addition, the role of type 1 fimbriae in biofilm formation has been recognized (42). Our study supports these data. Biofilm production in ST405 was strong and prevalence of different biofilm production was reported in ST131. In the present study, ST-361, ST-744, ST-101 and ST-4433 had only one virulence factor *fimH*, but the rest STs had more virulence factors. In previous studies,* E. coli *ST131 isolates had more antibiotic resistance profile, virulence factors and biofilm production. Also, it originates from phylogenetic group B2 (3) that is related epidemiologically to extraintestinal virulence (43) . According to our present findings, one of the ST-131 was resistant to all of the antibiotics except AK, and also it had all of the virulence genes and had moderate biofilms production. This study confirmed that ST-131 has become extensively disseminated in hospital. Also, the UPEC ST-131 strain is resistant to cephalosporins, aminoglycosides and trimethoprim/sulfamehoxazole, which is considered as serious threat to public health. In this study, ST complexes that are associated with UPEC were relatively uncommon in country.

In summary, we identified a high frequency of the ST131 clonal group and prevalence of antibiotic resistance, and virulence factor among UTIs in Kerman, Iran. These factors cause competitive advantage of this clonal group, supporting its rapid worldwide dissemination. These data show an important public health threat, which necessitate further studies to control the dissemination and risk factors for acquisition of the ST131 clonal group and other STs to make effective control. Our results suggest that several ST seem to be circulating in our region.

## Conclusion

As a study limitation there was only 20 isolates to detect ST. However, this study is the first report about dissemination of different STs lineage harboring *bla*_CTX-M-15 _among UPEC in Iran. We identified a high frequency of the ST131 clonal group, prevalence of antibiotic resistance, and virulence factor among UTIs. These data show an important public health threat and so further studies are needed to control the dissemination and risk factors for acquisition of the ST131 clonal group and other STs.
